# Rotational Dynamics of Organic Cations in Formamidinium Lead Iodide Perovskites

**DOI:** 10.1021/acs.jpclett.3c00185

**Published:** 2023-03-10

**Authors:** Rasmus Lavén, Michael M. Koza, Lorenzo Malavasi, Adrien Perrichon, Markus Appel, Maths Karlsson

**Affiliations:** † Department of Chemistry and Chemical Engineering, Chalmers University of Technology, SE-412 96 Göteborg, Sweden; ‡ Institut Laue-Langevin, 71 avenue des Martyrs, CS 20156, 38042 Grenoble cedex 9, France; § Department of Chemistry and INSTM, University of Pavia, Viale Taramelli 16, Pavia 27100, Italy; ∥ ISIS Facility, STFC Rutherford Appleton Laboratory, Didcot OX11 0QX, United Kingdom

## Abstract

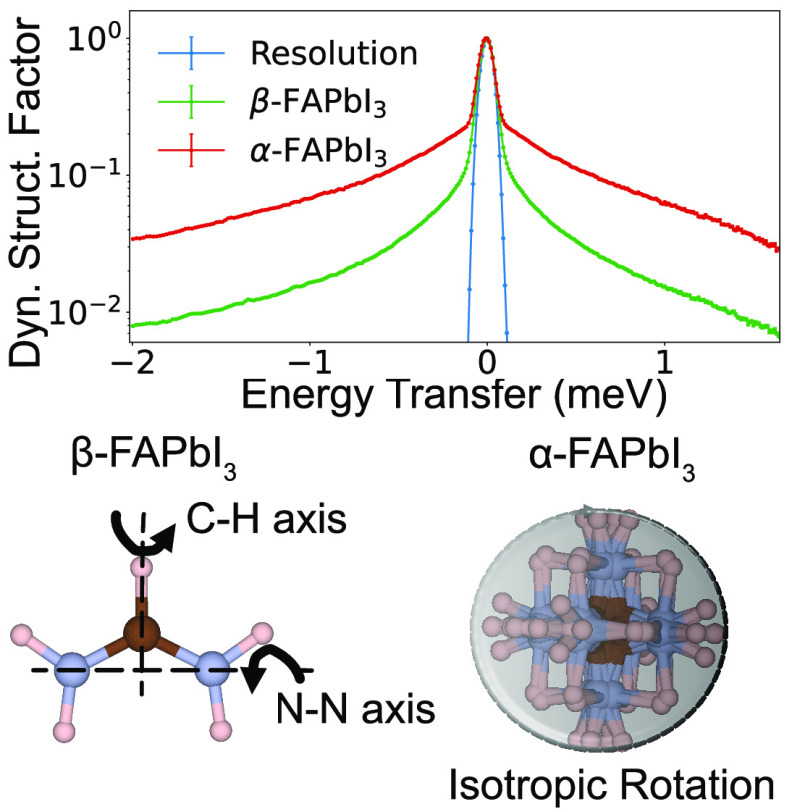

We report results from quasi-elastic neutron scattering studies on the rotational dynamics of formamidinium (HC[NH_2_]_2_^+^, FA) and methylammonium (CH_3_NH_3_^+^, MA) cations in FA_1–*x*_MA_*x*_PbI_3_ with *x* = 0 and 0.4 and compare it to the dynamics in MAPbI_3_. For FAPbI_3_, the FA cation dynamics evolve from nearly isotropic rotations in the high-temperature (*T* > 285 K) cubic phase through reorientations between preferred orientations in the intermediate-temperature tetragonal phase (140 K < *T* ⩽ 285 K) to an even more complex dynamics, due to a disordered arrangement of the FA cations, in the low-temperature tetragonal phase (*T* ⩽ 140 K). For FA_0.6_MA_0.4_PbI_3_, the dynamics of the respective organic cations evolve from a relatively similar behavior to FAPbI_3_ and MAPbI_3_ at room temperature to a different behavior in the lower-temperature phases where the MA cation dynamics are a factor of 50 faster as compared to those of MAPbI_3_. This insight suggests that tuning the MA/FA cation ratio may be a promising approach to tailoring the dynamics and, in effect, optical properties of FA_1–*x*_MA_*x*_PbI_3_.

Hybrid organic–inorganic perovskites (HOIPs) are currently attracting considerable attention because of their photovoltaic and photoluminescent properties and concomitant promise for use in both solar cells and light-emitting diodes.^1^ The prototypical HOIPs are methylammonium lead iodide (CH_3_NH_3_PbI_3_, MAPbI_3_) and formamidinium lead iodide (HC[NH_2_]_2_PbI_3_, FAPbI_3_), which feature optical band gaps nearly optimal for solar absorption.^2^ Various cation and/or anion substitutions are common means of altering the structure and photophysical properties of HOIPs.^3−7^ Beyond structural modifications, there is an increasing body of work that suggests that the dynamical nature of the organic cations plays an important role in the optical properties of HOIPs.^8−21^ Rotational organic cation dynamics have been invoked for explaining the formation of ferroelectric domains and surface ferroelectricity,^8^ exciton binding energy,^9^ hot carrier cooling,^22^ and charge carrier recombination rates^23−25^ in these types of materials.

The nature of rotational organic cation dynamics in HOIPs has been the subject of much theoretical and experimental research. Theoretically, the dynamics have been investigated using different computer models and approximations.^26^ The results have showcased various rotational motions of the organic cations, with characteristic relaxation times in the range of 1–100 ps, but the results have sometimes been conflicting, which reflects the complexity of the problem.^27^ Experimentally, the dynamics can be probed by quasi-elastic neutron scattering (QENS), which has been demonstrated for MAPbI_3_,^8−10,14^ and the related materials MAPBr_3_^15,28^ and MAPbCl_3_.^29,30^ In MAPbI_3_, the MA cation has been shown to exhibit different dynamics depending on temperature due to the different phases present. In the high-temperature (*T* > 330 K) cubic phase (*Pm*3̅*m*), the MA cations undergo fully isotropic rotational motions, whereas in the room-temperature tetragonal phase (*I*4/*mcm*) rotations occur with preferred orientations related to the 4-fold (*C*_4_) symmetry around the crystallographic *c* axis.^9^ In the low-temperature (*T* < 170 K) orthorhombic phase (*Pnma*), only 3-fold (*C*_3_) rotations of the CH_3_ and/or NH_3_ group around the C–N axis persist (cf. Figure 1).^9^ A similar dynamical picture has been obtained for MAPBr_3_^15,28^ and MAPbCl_3_,^29,30^ with the halide anion affecting the relaxation times and activation energies of the dynamics.

**Figure 1:**
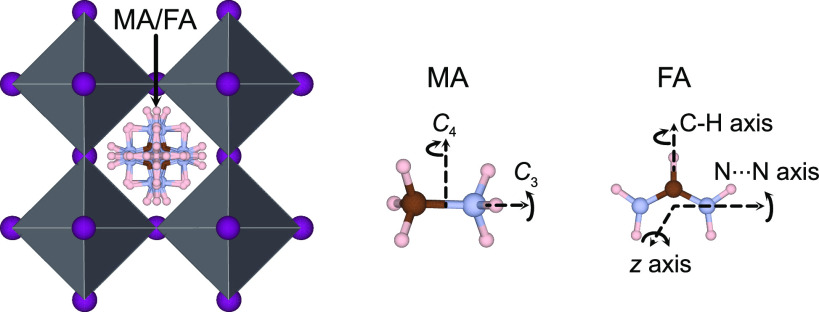
Illustration of the various possible rotational modes of MA^9,39^ and FA.^13,31,35^ I, C, N, and H atoms are illustrated as purple, brown, blue, and pink spheres, respectively. The Pb atoms lie within black octahedra. The image was produced using VESTA.^40^

In contrast to the MA-based HOIPs, the dynamical nature of FA cations in HOIPs has been studied much less and to the best of our knowledge no QENS studies have been reported on FAPbI_3_, but recently, QENS studies were performed on the related material FAPbBr_3_.^31,32^ In particular, the study by Sharma et al.^31^ indicated that the FA cation dynamics are isotropic in nature, with a continuously increasing number of FA cations participating in the dynamics with increasing temperature from 100 to 350 K. However, other studies on FAPbBr_3_ and FAPbI_3_, based on temperature-dependent photoconductivity,^32^ nuclear magnetic resonance (NMR),^11,13,32−34^ optical spectroscopy,^35^ and molecular dynamics simulations,^36^ have indicated that the FA cation dynamics are anisotropic and feature preferred reorientations around the N···N axis.^13^ Consequently, a consensus regarding FA cation dynamics in HOIPs is missing.

The stable structure of FAPbI_3_ at room temperature is a nonperovskite hexagonal structure (δ-phase, *P*6_3_/*mmc*), but a metastable cubic (α) perovskite phase (*Pm*3̅*m*) can be stabilized at room temperature for several days by heating to above the δ−α phase transition at around 410 K.^4,37,38^ Upon cooling the α-phase, the structure transforms to a tetragonal (β) phase (*P*4/*mbm*) for 140 K ≲ *T* ≲ 285 K and to yet another tetragonal (γ) phase for *T* ≲ 140 K.^2,38,41−43^

In this work, using QENS, we show that the FA cation dynamics evolve from nearly isotropic rotations in the high-temperature (*T* ≳ 285 K) cubic phase through rotational motions between preferred orientations in the intermediate-temperature tetragonal phase (140 K ≲ *T* ≲ 285 K) to even more complex dynamics in its low-temperature tetragonal phase (*T* ≲ 140 K). Additionally, we show that the dynamics in the mixed-cation system FA_0.6_MA_0.4_PbI_3_, a composition that has been selected due to its excellent solar-cell efficiency,^44^ feature strikingly different cation dynamics with respect to the respective single-cation systems. Tuning of the MA/FA concentration ratio thus represents a promising gateway to tune the dynamic and optical properties of the FA_1–*x*_MA_*x*_PbI_3_ system.

Figure 2 (a) shows data of an elastic fixed window scan (EFWS) and inelastic fixed window scans (IFWSs) at 4 and 10 μeV of FAPbI_3_, as measured on IN16B upon cooling the cubic α-phase of FAPbI_3_ from *T* = 300 to 2 K. We observe no major change when passing through the cubic-to-tetragonal (α–β) phase transition at around 285 K,^38^ indicating that the FA cation dynamics are too fast to be observed by IN16B in these two phases. Upon approaching the transition temperature of the tetragonal β-phase to the tetragonal γ-phase at around 140 K,^42^ the elastic intensity starts to increase more rapidly, indicating a significant slowing down of the FA dynamics when entering the γ-phase, making the dynamics accessible in the time window of IN16B (∼6–300 ps). Note that the inelastic intensities show broad maxima near the β–γ phase-transition temperature. This is especially pronounced for the IFWS taken at 4 μeV, which shows almost a plateau between *T* = 140 and 90 K. This suggests that several dynamical processes, with slightly different relaxation times and activation energies, are taking place within the measured time window.

**Figure 2:**
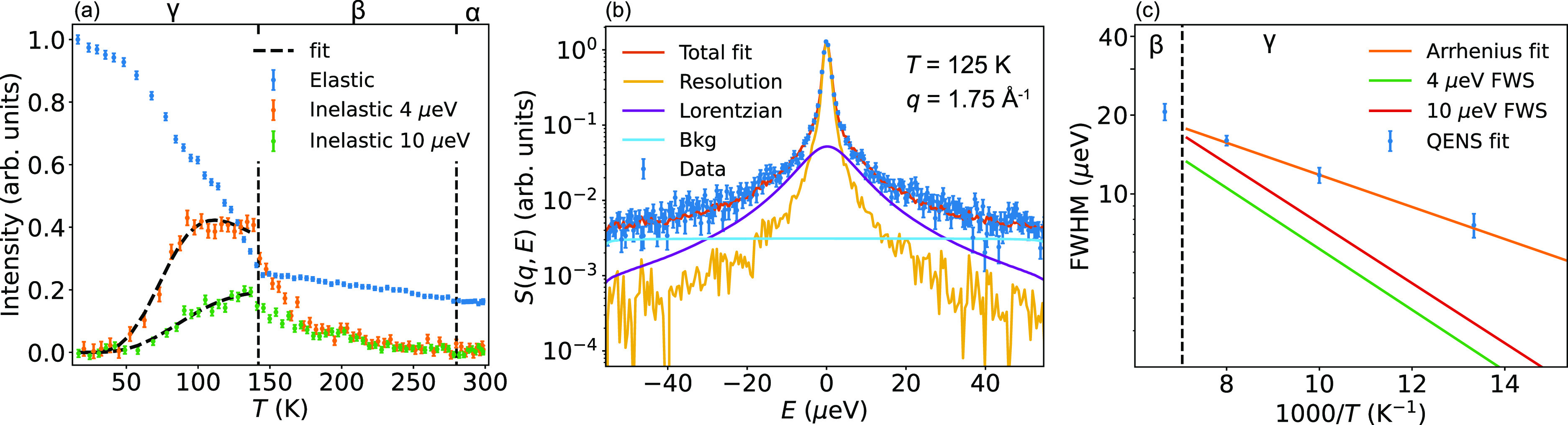
QENS data of FAPbI_3_ measured on IN16B. (a) EFWS and IFWSs summed over all measured *q* values as a function of temperature. The EFWSs intensity is normalized to a maximum value of unity, and the IFWS intensities are multiplied by a factor of 15 for increased visibility. An elastic contribution was subtracted from the IFWS by determining the relative intensity at 4 and 10 μeV in the 2 K QENS spectrum. (b) Fit of *S*(*q*, *E*) measured at *T* = 125 K and *q* = 1.75 Å^–1^. (c) *T* dependence of the quasi-elastic line width from fits to the IFWS and QENS spectra. The solid orange line represents an Arrhenius fit with an activation energy of 13 meV, and the dashed black line indicates the γ-to-β phase transition. The data point at 150 K is in the β phase and is not included in the Arrhenius fit.

For a single relaxational process with an Arrhenius behavior of the relaxation time, the inelastic scattering intensity is given by the following expression:^45^
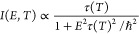
1Here, τ(*T*) = τ_0_ exp(*E*_a_/*k*_B_*T*) specifies the relaxation time, where *E*_a_ is the activation energy, τ_0_ is an exponential prefactor, *T* is the temperature, ℏ is the reduced Planck constant, and *k*_B_ is the Boltzmann constant. We fitted the inelastic intensities as a function of temperature in the γ-phase to the expression in eq 1. By doing so, *E*_a_ takes on values of 23 ± 4 and 23 ± 3 meV for the 10 and 4 μeV data, respectively. Thus, the data at different energies give consistent results for *E*_a_.

The relatively slow dynamics in the tetragonal γ-phase of FAPbI_3_ were further analyzed in terms of the dynamical structure factor, *S*(*q*, *E*). *S*(*q*, *E*) was fitted to a function of the form
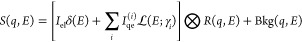
2Here, *I*_el_ and *I*_qe_ are the elastic and quasi-elastic intensities, respectively,  represents Lorentzian functions with line widths (fwhm) γ_*i*_, *R*(*q*, *E*) is the resolution function of the instrument, and Bkg(*q*, *E*) = *a*(*q*) + *b*(*q*)·*E* is a sloping background with *a* and *b* as constants. Our analysis showed that only one Lorentzian function (*i* = 1) was needed to adequately account for the quasi-elastic part for all measured temperatures and *q* values. Figure 2 shows, as an example, the QENS spectrum as measured at *T* = 125 K and *q* = 1.75 Å^–1^. The line width showed no (within error) dependence on *q* (Figure S2), which suggests that the quasi-elastic scattering is related to localized motions of the FA cations. Figure 2 (c) shows a plot of the *q*-averaged line widths as a function of temperature. Included in the plot is the predicted Arrhenius temperature dependence of the line width as extracted from the IFWSs. Fitting to an Arrhenius dependence suggests that the dynamics are characterized by an activation energy of 13 ± 4 meV, which is much lower than what was extracted from the fit to the IFWSs (about 23 meV). For comparison, the corresponding neutron data for MAPbI_3_ show much better agreement with respect to the QENS line shape and FWS data; see the QENS data on MAPbI_3_, which are summarized in Figures S3–S5. This shows that the MA cation dynamics in the orthorhombic phase of MAPbI_3_ are well described by a single dynamical component that can be adequately modeled as a Lorentzian function. The worse agreement between the IFWS fit and the width extracted from the fitting of the QENS spectra for FAPbI_3_ suggests that the FA cation dynamics are more complex than the MA dynamics in the γ-phase and that they are not well described by a single relaxational process. This is reasonable since, while the MA cations in the low-temperature orthorhombic phase of MAPbI_3_ are more ordered,^46^ the γ-phase of FAPbI_3_ is believed to be locally disordered with potentially no long-range order of the FA cations.^13,38,47^

Faster time-scale dynamics (∼0.1–13 ps) in the tetragonal β- and cubic α-phases of FAPbI_3_ were investigated on IN6 and IN5. Figure 3 (a) shows the QENS spectra of FAPbI_3_ at *q* = 1.54 Å^–1^ in the tetragonal β-phase (200 K) and cubic α-phase (300 K), as measured on IN5. In the β- and α-phases, two Lorentzian functions were required to model the quasi-elastic scattering, and we thus fitted *S*(*q*, *E*) to the model function in eq 2 with fixed *q*-independent line widths of the two Lorentzians. The line width follows no clear Arrhenius dependence over the full temperature range [Figure 3 (c)]. However, we note a stronger temperature dependence in the cubic α-phase than in the tetragonal β-phase [Figure 3 (b)]. This suggests a higher activation energy for molecular rotations in the cubic α-phase. In the tetragonal γ-phase, at 100 K, a weak QENS signal was observed on IN5, which could be modeled using a single Lorentzian function with a line width of about 0.2 meV. We note that this line width corresponds to much faster dynamics than observed on IN16B at the same temperature. This strengthens the claim that there is a wide distribution of relaxational times in the tetragonal γ-phase, as most dynamics at 100 K were found to be accessible on IN16B, which probes time scales in the range of 6–300 ps. Still, there is a small fraction of FA cations with faster dynamics even below 100 K, which could be accessed on IN5.

**Figure 3:**
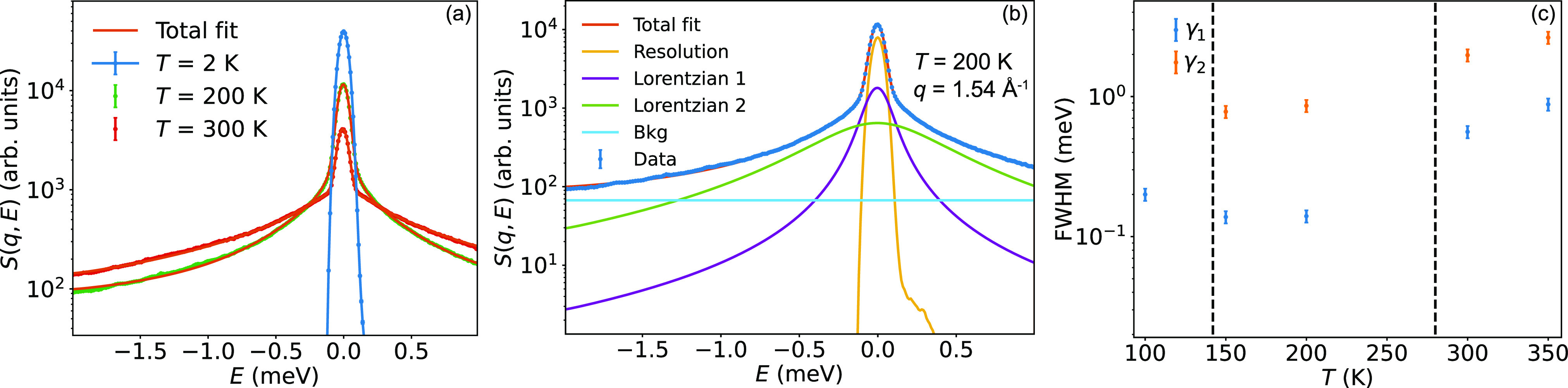
QENS data of FAPbI_3_ measured on IN5. (a) Quasielastic line shape at *q* = 1.54 Å^–1^ in the β-phase (200 K) and α-phase (300 K), together with total fits using two Lorentzian functions to describe the QENS. (b) Fit with separate fitting components plotted as *S*(*q*, *E*) for FAPbI_3_ measured at *T* = 200 K on IN5. (c) Temperature dependence of the quasi-elastic line widths (fwhm) of the two fitted Lorentzians. The dashed lines mark the phase-transition temperatures.

Information about the spatial geometry of the observed dynamics was obtained by the analysis of the elastic incoherent structure factor (EISF), defined as EISF = *I*_el_/(*I*_el_ + ∑_*i*_*I*_qe_^(*i*)^). Figure 4 shows the experimentally determined EISF for the temperatures *T* = 100, 150, 200, 300, and 350 K, together with geometrically feasible models of localized FA cation dynamics. In the cubic α-phase, at *T* = 300 and 350 K, the EISF can be adequately approximated with a model that describes the FA cation dynamics as isotropic rotation. The minimum in the EISF occurs at around *q* = 1.8 Å^–1^. This is in excellent agreement with the effective FA molecular radius of 1.855 Å,^31^ which thus indicates that the whole molecule rotates in the cubic phase. We observe that the data show slightly larger elastic scattering at the *q* values where the minimum occurs as compared to the isotropic model, which might be indicative of the fact that there are some slight preferences of the FA orientations also in the cubic phase. However, such a small amount of extra elastic scattering may originate from a small part of the quasi-elastic scattering signal lying in the background in the fitting of the neutron scattering spectra and is thus hard to estimate experimentally. Note that there is a comparison to a model describing jump diffusion among 12 different jump locations, where the C–H bond can point toward any of the cube faces, and for each of these 6 directions, there are 2 possible orientations of the N···N axis, as suggested by Weller et al.,^39^ that do not describe the data in any better way. In addition, the ratio of the two line widths of the two fitted Lorentzians is about 3.5 at 300 K, which is in good agreement with what is expected (3) from the isotropic rotational jump diffusion model.^48^ Isotropic rotations in the cubic phase of FAPbI_3_ are also in agreement with a previous neutron diffraction study,^41^ which indicated no preferred orientations of the FA cations, and a recent NMR study,^34^ which shows that the relaxation times for all FA cation rotations are less than 2 ps.

**Figure 4:**
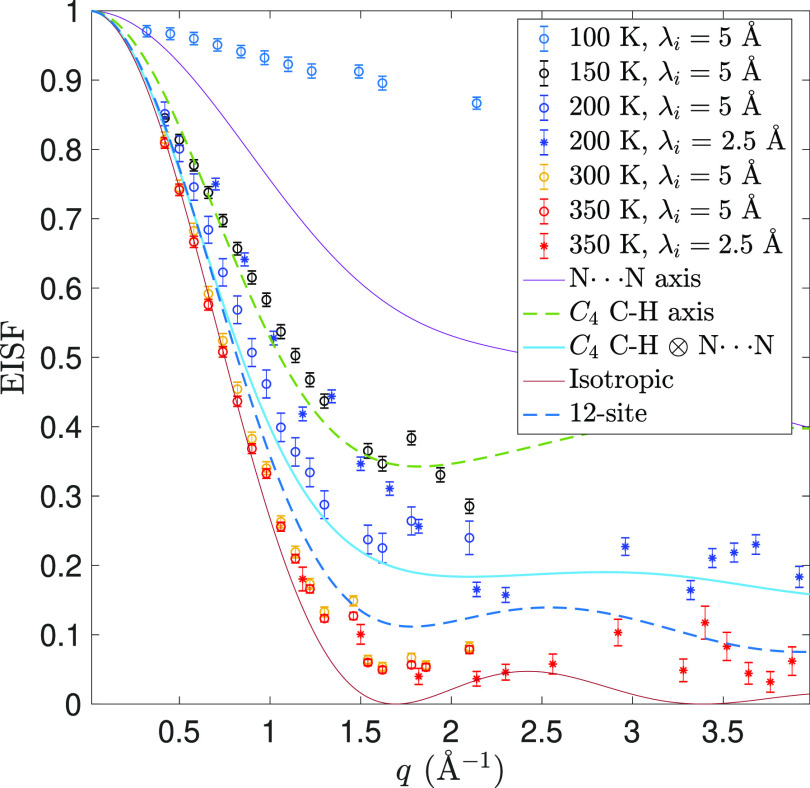
EISF of FAPbI_3_ extracted from fits to the QENS data from IN5 at various temperatures. The data is compared to several jump diffusion models that describe localized reorientational motions of the FA cation. Data points around 1.85 Å^–1^ are removed for the tetragonal phases due to a Bragg peak.

In the tetragonal β-phase, at *T* = 200 and 150 K, the minimum in the EISF is shifted to higher *q*, to about 2.5 Å^–1^ at 200 K. This indicates that the effective jump distance is smaller than in the cubic α-phase, which could occur if there are some strong preferred orientations of the FA cations and/or rotations occur only around some specific axes. In addition, the ratio of the two QENS line widths is in the range of 5 to 6, which is much larger than in the cubic phase, thus suggesting that the two Lorentzians might instead be related to two different rotational modes. Weber et al.^38^ showed that, in the tetragonal β-phase (*P*4/*mbm* space group), the FA cation is located on Wyckoff site 2*c*, which has *D*_2*h*_ point group symmetry that allows for 2-fold (*C*_2_) rotations of the FA cation around its three principal axes. In this structure, the FA cations are disordered amongst four sites, which mainly involve rotations around the N···N axis. Furthermore, Fabini et al.^13^ claimed, based on NMR and molecular dynamics simulations, that the dominant FA cation dynamics are associated with reorientations around the N···N axis, in all phases of the material. A comparison of the experimentally determined EISF with jump-diffusion models that describe rotations among the four sites in the structural model of the β-phase of FAPbI_3_ suggested by Weber et al.^38^ (N···N in Figure 4) shows that more quasi-elastic scattering is observed than this model predicts. This indicates that there are additional relaxational dynamics at 200 K. The data can be approximately described by a jump diffusion model consisting of a 4-fold rotation around the C–H axis and jumps around the N···N axis, suggesting that the main axes of rotation in the tetragonal β-phase are the N···N axis and the C–H axis. This is also in agreement with the recent NMR study^34^ that shows that the relaxation time is indeed the shortest for rotation around these specific axes. The data at *T* = 150 K are merely a shift to higher elastic scattering from the *T* = 200 K data, with no significant change in the *q* dependence, which suggests that the geometry of the dynamics is the same at *T* = 200 and 150 K. The higher elastic intensity at *T* = 150 K may be explained by, at this temperature, some of the FA cations being immobile on the probed time scale. These immobile FA cations rather contribute to an enhanced elastic scattering in the measurement. At even lower temperature, in the tetragonal γ-phase at *T* = 100 K, there is only a small amount of quasi-elastic scattering, and the EISF decays almost linearly to a value of about 0.87 at *q* ≈ 2 Å^–1^. This suggests that only a small portion of the FA cations are dynamically active within the experimentally probed time scale (0.1–10 ps) at this temperature. Presumably, these mobile FA cations may be related to specific local environments, as the tetragonal γ-phase is believed to be locally disordered with no long-range ordering of the FA cations.^38^

It is interesting to note the contrasting dynamics observed here for FAPbI_3_ compared to a previous QENS study of FA cation dynamics in the related material FAPbBr_3_.^31^ In that study, it was found that the FA cations perform isotropic rotations independently of temperature and crystallographic phase. However, one may note that the explored *q* and *E* ranges were relatively limited compared to our measurements. This makes it hard to separate different models of the EISF and to capture all quasi-elastic signals in the fitting of the QENS spectra. Contrastingly, we observe FA cation dynamics which are dependent on both temperature and the symmetry of the surrounding perovskite cage. Sharma et al.^49^ observed similar dynamics in FAPbCl_3_, where the only observed rotational mode in the low-temperature phase was 2-fold rotation around the C–H axis. This suggests that the geometry of the FA cation dynamics is intrinsically determined by the surrounding perovskite cage geometry and halide anion.

The crystal structure of the mixed-cation material FA_0.6_MA_0.4_PbI_3_ shows similar phase behavior upon cooling as FAPbI_3_. It undergoes a phase transition from a high/room-temperature cubic α-phase to an intermediate-temperature tetragonal phase at around 270 K (*P*4/*mbm*, β-phase) and to another tetragonal phase at around 200 K.^50^Figure 5 (a) shows *S*(*q*, *E*) for FA_0.6_MA_0.4_PbI_3_, as measured at *T* = 250 K and *q* = 1.61 Å^–1^ on IN6. Similar to the *S*(*q*, *E*) for FAPbI_3_ and MAPbI_3_, the *T* = 200 K data are characterized by a large quasi-elastic component, and two Lorentzian functions were needed to describe the quasi-elastic scattering. The two Lorentzians exhibit average line widths of around 1.3 and 0.18 meV, respectively, at *T* = 250 K, and the line widths are essentially *q*-independent. One should note, however, that each Lorentzian cannot be simply related to a single dynamical process since, based on the results of FAPbI_3_ and MAPbI_3_, the FA cation is expected to have at least one dynamical process and the MA cation is expected to have at least two dynamical processes under the measurement conditions. Because of the fact that they occur on similar time scales (cf. Figures 3 and S5), they may be hard to separate experimentally. Therefore, the activation energies of 106 meV for the narrower Lorentzian and 13 meV for the broader Lorentzian function, as extracted from the temperature dependence of the respective line width [Figure 5 (b)], represent average, or “apparent”, values. The 13 meV activation energy is related to the broader Lorentzian function which mainly involves contributions from *C*_3_ rotations of MA (as will be shown below; cf. Figure 5 (e)). Note that this value is much lower than the corresponding value in the orthorhombic phase of MAPbI_3_ (48 meV).^14^ The activation energy of 106 meV is related to the more narrow Lorentzian and contains contributions from the rotations of both MA and FA.

**Figure 5:**
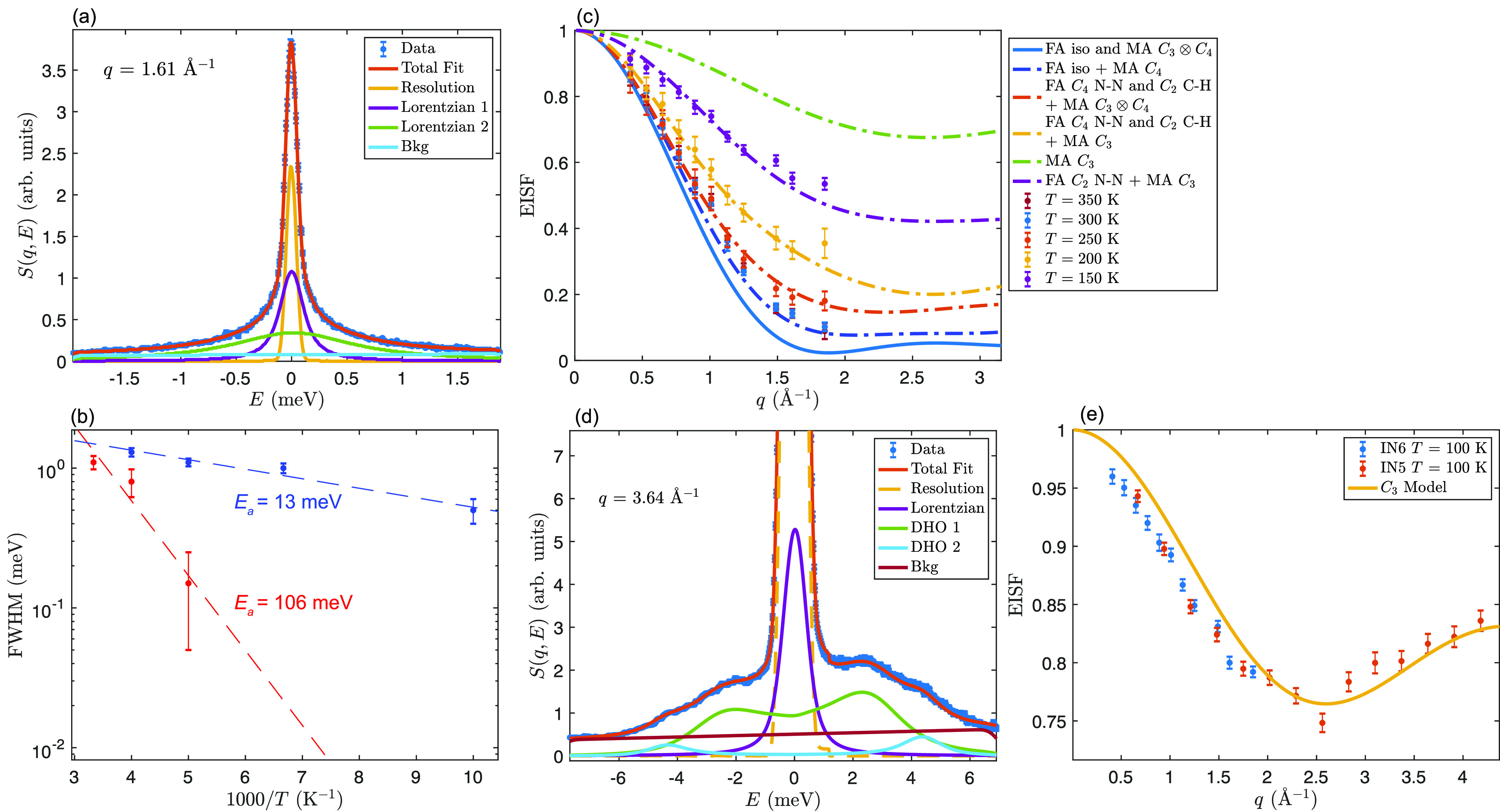
QENS data of FA_0.6_MA_0.4_PbI_3_ measured on IN6 and IN5. (a) Fit to the quasi-elastic scattering of FA_0.6_MA_0.4_PbI_3_ as measured on IN6 at *T* = 250 K. (b) Arrhenius plot of the quasi-elastic line width for the two fitted Lorentzians of FA_0.6_MA_0.4_PbI_3_. The fast process (in blue) is assigned to the *C*_3_ rotations of MA and yields an activation energy of about 13 meV. The slower process (in red) is assigned to full molecular rotations of both MA and FA and yields an activation energy of about 106 meV. (c) Extracted EISF for different temperatures measured on IN6. The data are compared to various jump diffusion models that describe localized reorientational motions of the MA and FA cations. (d) Fit of *S*(*q*, *E*) for FA_0.6_MA_0.4_PbI_3_ measured at *T* = 100 K using an incident neutron wavelength of 2.5 Å on IN5. (e) EISF of FA_0.6_MA_0.4_PbI_3_ probed with 2.5 Å (IN5) and 5.1 Å (IN6) incident neutron wavelengths at *T* = 100 K. The data are fitted to the *C*_3_ model of MA with an immobile fraction of 0.71, which is close to what is expected from the stoichiometry.

Crucially, even though all dynamical processes for FA_0.6_MA_0.4_PbI_3_ cannot be separated, the EISF can be robustly estimated from the QENS fit, as it is not as sensitive to the details of the fitting but only to the extracted quasi-elastic and elastic intensities. Figure 5 (c) shows the EISF of FA_0.6_MA_0.4_PbI_3_ at *T* = 150, 200, 250, 300, and 350 K. The EISFs are compared to models that are based on a superposition of the reorientational dynamics in MAPbI_3_ and FAPbI_3_, respectively, with the contributions from FA and MA weighted by the stoichiometry of the mixed-cation sample, i.e., 40% MA and 60% FA. In the cubic α-phase, at *T* = 350 and 300 K, the EISFs are best described by a model that considers *C*_4_ rotations of the MA together with isotropic rotation of FA. Note that the *C*_3_ rotational mode of MA (cf. Figure 1) is most likely too fast to be observed for FA_0.6_MA_0.4_PbI_3_ in the cubic α-phase and thus its intensity contributes to the background (as will be shown below). In the tetragonal β-phase, at *T* = 250 K, the EISF is best described by a model in which the FA cation undergoes *C*_4_ rotation around the N···N axis together with *C*_2_ rotation around the C–H axis and where the MA cation undergoes *C*_3_ ⊗ *C*_4_ rotations. Such *C*_3_ ⊗ *C*_4_ rotational dynamics have been previously observed in the tetragonal β-phase of MAPbI_3_.^9^ Upon lowering the temperature to *T* = 200 K, the dynamics of the FA cations persist, but now, the MA cations undergo only *C*_3_ rotations. At the lowest temperature, *T* = 150 K, the data can be adequately described by *C*_3_ rotations of the MA cations together with *C*_2_ rotations of the FA cations around the N···N axis. For *T* ≥ 150 K, it can thus be concluded that the dynamics of the FA and MA cations in FA_0.6_MA_0.4_PbI_3_ can be described by a combination of the same type of FA and MA cation dynamics as observed in the respective pure end-member compounds. Note that the EISF of FA_0.6_MA_0.4_PbI_3_ can be equally well approximated by an isotropic-rotation model with a fraction of immobile cations. However, we have no physical explanation for using such a model, and the observed fast motion can be accurately assigned to the MA *C*_3_ cation rotations, as described below.

In order to confirm our results so far, we also performed an experiment on IN5 over an extended *q* range up to about 4 Å^–1^ using an incident neutron wavelength of 2.5 Å. Because of the extension of the energy range, these measurements feature a significant overlap between QENS and inelastic scattering from phonons. To analyze this data, *S*(*q*, *E*) was fitted to one or several quasi-elastic Lorentzians and two damped harmonic oscillator functions describing the phonons.^51^Figure 5 (e) compares the EISF of FA_0.6_MA_0.4_PbI_3_ at *T* = 100 K as extracted from measurements using neutrons with incident wavelengths of both 5.1 Å (IN6) and 2.5 Å (IN5). The data are compared to a model that assumes that only the methyl/ammonia group of the MA cations performs the *C*_3_ rotations. As can be seen in Figure 5 (e), the extended *q*-range allows for an unequivocal assignment of the observed dynamics to *C*_3_ rotations of the MA cation. The *C*_3_ rotations in FA_0.6_MA_0.4_PbI_3_ have an average relaxation time of about 3 ps at 100 K, which is drastically faster than that in MAPbI_3_ at the same temperature (136 ps). This may be related to the absence of a phase transition to an orthorhombic phase upon cooling FA_0.6_MA_0.4_PbI_3_ and the increased disorder.

Our studies reveal distinct differences in the organic cation dynamics for the mixed-cation system FA_0.6_MA_0.4_PbI_3_ compared to the pure end-member compounds. Even though there are some differences in the low-temperature phases, the organic cation dynamics appear in the high-temperature phases to be quite similar for all samples. In particular, in the cubic α-phase at 350 K, the average relaxation time (calculated as 2ℏ/γ) is about 1.4, 1.5, and 1.1 ps for MAPbI_3_, FAPbI_3_, and FA_0.6_MA_0.4_PbI_3_, respectively. Below 280 K, MAPbI_3_ and FAPbI_3_ display quite different crystal structures, while at higher temperatures, they all exhibit a cubic structure. We note that this is in agreement with our recent inelastic neutron scattering and first-principles simulation study of FA_1–*x*_MA_*x*_PbI_3_^52^ showing that MA doping of FAPbI_3_ leads to an increased level of hydrogen bonding between the FA cations and the lead iodide framework as a result of cage deformation, which at the same time leads to a weakening of the MA–cage interactions. Further, the QENS results show that this leads to drastically faster MA cation dynamics in the low-temperature phase of FA_0.6_MA_0.4_PbI_3_. Our results are also in agreement with a recent QENS study of FA_0.125_MA_0.875_PbI_3_, showing a complete suppression of the FA cation dynamics and faster MA cation dynamics in this material.^53^ However, we show that this faster MA cation dynamics is most likely related to the *C*_3_ methyl/ammonia group rotations and not to the rotations of the whole MA cation.

To conclude, for FAPbI_3_, the dynamics of the FA cations evolve from nearly isotropic motions in the cubic (α) phase (*T* ≳ 285 K), through reorientational motions between preferred orientations with the main rotational axes being the N···N and C–H axes in the tetragonal (β) phase (140 K ≲ *T* ≲ 285 K), to even more complex dynamics, due to a disordered arrangement of the FA cations, in the tetragonal (γ) phase (*T* ≲ 140 K). In comparison, FA_0.6_MA_0.4_PbI_3_ exhibits considerably different dynamics with respect to the respective single-cation systems, suggesting that detailed mixing of the cation ratio in mixed-cation systems offers a novel route to tailoring the dynamics and potentially the optical properties of metal halide perovskites.

## Experimental Details

The QENS experiments were performed on three different instruments: the two direct-geometry time-of-flight spectrometers IN5^54^ and IN6^55^ and the backscattering spectrometer IN16B^56^ at the Institut Laue-Langevin (ILL), Grenoble, France. After general and instrument-specific data reductions, which are briefly outlined in the following text, the computed response function in each experiment is the dynamical structure factor, *S*(*q*, *E*), where *q* and *E* are the moduli of the wavevector transfer and energy transfer, respectively. The complementarity in using IN5, IN6, and IN16B is that they allow us to probe different parts of (*q*, *E*) space with different energy resolutions, meaning that information about the dynamics over a large range of time and length scales can be obtained.

IN6 was set up using an incident neutron wavelength of 5.1 Å, which yields an *E* resolution at the full width at half-maximum (fwhm) of 70 μeV. The accessible *q* range was ∼0.3–2 Å^–1^ at the elastic line. On IN5, incident neutron wavelengths of 5 and 2.5 Å were used. The use of 5 Å wavelength neutrons yields an *E* resolution at fwhm of 0.1 meV and a *q* range of ∼0.3–2.2 Å^–1^ at the elastic line. The use of 2.5 Å wavelength neutrons yields an *E* resolution at fwhm of 0.62 meV and a *q* range of ∼0.5–4 Å^–1^ at the elastic line. For both the IN6 and IN5 data, standard data reduction included normalization to a vanadium standard, background subtraction (empty sample cell), and correction of the energy-dependent efficiency of the detectors. Measurements were taken in the temperature range of 2–350 K, and the 2 K spectra were used as a resolution function in the data analysis. Data reductions were done with the LAMP software.^57^

IN16B was set up using an incident neutron wavelength of 3.275 Å and with Si(311) analyzer crystals. With this setup, the instrument yields an *E* resolution at fwhm of ∼2 μeV and an accessible *E* range of ±56 μeV. The accessible *q* range was 1.5–3.5 Å^–1^. In addition to the QENS measurements, measurements of the elastic and inelastic intensities upon temperature variation, so-called elastic and inelastic fixed window scans (EFWS and IFWS, respectively),^45^ were recorded upon cooling from *T* = 300 to 2 K. The inelastic intensities were probed at 4 and 10 μeV, respectively. Measurements were taken in the temperature range of 2–150 K with the 2 K spectra used as a resolution function in the data analysis. Data reductions were performed with the Mantid software.^58^

The samples, FA_1–*x*_MA_*x*_PbI_3_ (*x* = 0.0, 0.4 and 1.0) powders, were held inside rectangular (IN6) or annular (IN5, IN16B) aluminum sample holders. However, FAPbI_3_ was annealed *ex situ* at 438 K for 2 h directly prior to each of the measurements to form the metastable cubic (α) perovskite phase.^37^ The cubic structure was confirmed by the absence of any hexagonal Bragg peaks as seen in the energy-integrated neutron data on IN5 (Figure S1). The samples are the very same ones as used in our previous structural study by inelastic neutron scattering,^52^ and the details of the synthesis and characterization are reported in ref (4).

## Data Availability

Access to the neutron scattering data is provided according to the ILL data policies.^59,60^

## Supporting Information Available

Energy integrated (diffraction) data for FAPbI_3_; QENS data on MAPbI_3_; fits to QENS spectra; additional QENS data on FAPbI_3_ and FA_0.6_MA_0.4_PbI_3_; and details on the EISF models for FAPbI_3_ (PDF)
