# Supplementary material for: Rotational Dynamics of Organic Cations in Formamidinium Lead Iodide Perovskites
Source: J Phys Chem Lett. 2023 Mar 10;14(11):2784–91. doi: 10.1021/acs.jpclett.3c00185 (PMC10041645; doi:10.1021/acs.jpclett.3c00185)
Supplement: Supplementary file 1 — jz3c00185_si_001.pdf [file jz3c00185_si_001.pdf]

Supporting information:

Rotational Dynamics of Organic Cations in Formamidinium Lead Iodide Perovskites

Rasmus Lavén,[†] Michael M. Koza,[‡] Lorenzo Malavasi,[¶] Adrien Perrichon,[§] Markus Appel,[‡] and Maths Karlsson[*,†]

†*Department of Chemistry and Chemical Engineering, Chalmers University of Technology, SE-412 96 Göteborg, Sweden.*

‡*Institut Laue-Langevin, CS 20156, 38042 Grenoble Cedex 9, France.*

¶*Department of Chemistry and INSTM, University of Pavia, Viale Taramelli 16, Pavia 27100, Italy.*

§*ISIS Facility, Rutherford Appleton Laboratory, Harwell Oxford, Didcot, Oxfordshire OX11 0QX, United Kingdom.*

E-mail: maths.karlsson@chalmers.se

S1. Additional data on $FAPbI_3$ and $FA_{0.6}MA_{0.4}PbI_3$

S1.1 Energy integrated scattering

Figure S1 shows the energy integrated data on IN5 for $FAPbI_3$. There is no notable intensity at the expected positions of the Bragg peaks for the δ-phase of $FAPbI_3$, which shows that the sample was in pure perovskite phase during all neutron measurements.

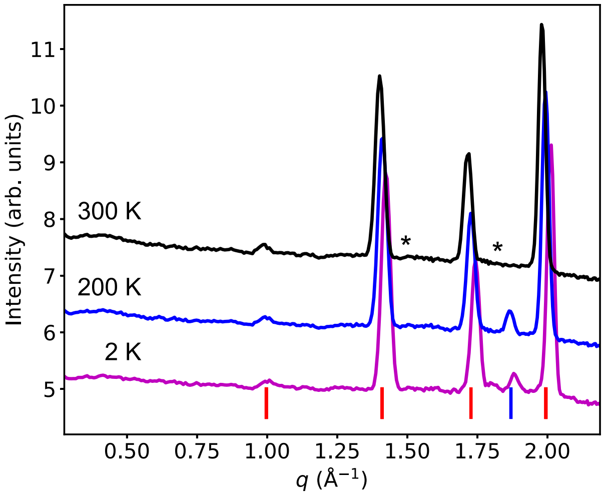

Figure S1: Energy integrated data for $FAPbI_3$ as measured on IN5 using an incident neutron wavelength of 5 Å. The data for different temperatures have been offset along the vertical axis for increased visibility. The red and blue ticks mark Bragg positions for the cubic and tetragonal phases, respectively. The asterisks mark the expected positions for the two strongest Bragg peaks of the δ-phase of $FAPbI_3$ at 300 K.[S1]

S1.2 Fitting of the QENS spectra of $FAPbI_3$

Figure S2 shows fits to the QENS spectra for different temperatures measured on IN16B together with the extracted quasielastic linewidth (FWHM) as a function of q and T.

S1.3 Fitting of the high q and E domain for $FAPbI_3$ & $FA_{0.6}MA_{0.4}PbI_3$

When using an incident neutron wavelength of 2.5 Å the probed energy range overlaps with the energy range of phonons, which appear for energies > 3 meV. For the analysis of the quasielastic part, $S(q, E)$ was fitted to the following model function which describes both the QENS and phonon contributions:

$$S(q, E) = S_{\text{QENS}}(q, E) + S_{\text{phonon}}(q, E). \tag{1}$$

The quasielastic part was described as before:

$$S_{\mathrm{QENS}}(q,E) = \left[I_{\mathrm{el}}\,\delta(E) + \sum_i I_{\mathrm{qe}}^{(i)}\,\mathcal{L}(E;\gamma_i) \right] \otimes R(q,E) \\ + \mathrm{bkg}(q,E), \tag{2}$$

with the linewidth fixed to the value from the fit of the IN6 data. The phonon part was described as a sum of damped harmonic oscillators:

$$S_{\mathrm{phonon}}(q,E) = \sum_i \frac{[n(\omega)+1]Z(q)4\omega\Gamma_i/\pi}{[(\omega-\omega_i)^2+\Gamma_i^2]\,[(\omega+\omega_i)^2+\Gamma_i^2]}, \tag{3}$$

where $n(\omega)$ is the Bose-Einstein population factor, $Z(q)$ is the one-phonon structure factor, Γ_i is a damping factor, and ω_i is the phonon frequency.[S2] Above $\sim$ 200 K, these phonons are overdamped, and, therefore, we limited the fitted energy interval to $\pm$4 meV, in order to capture their contribution in the fitted background function.

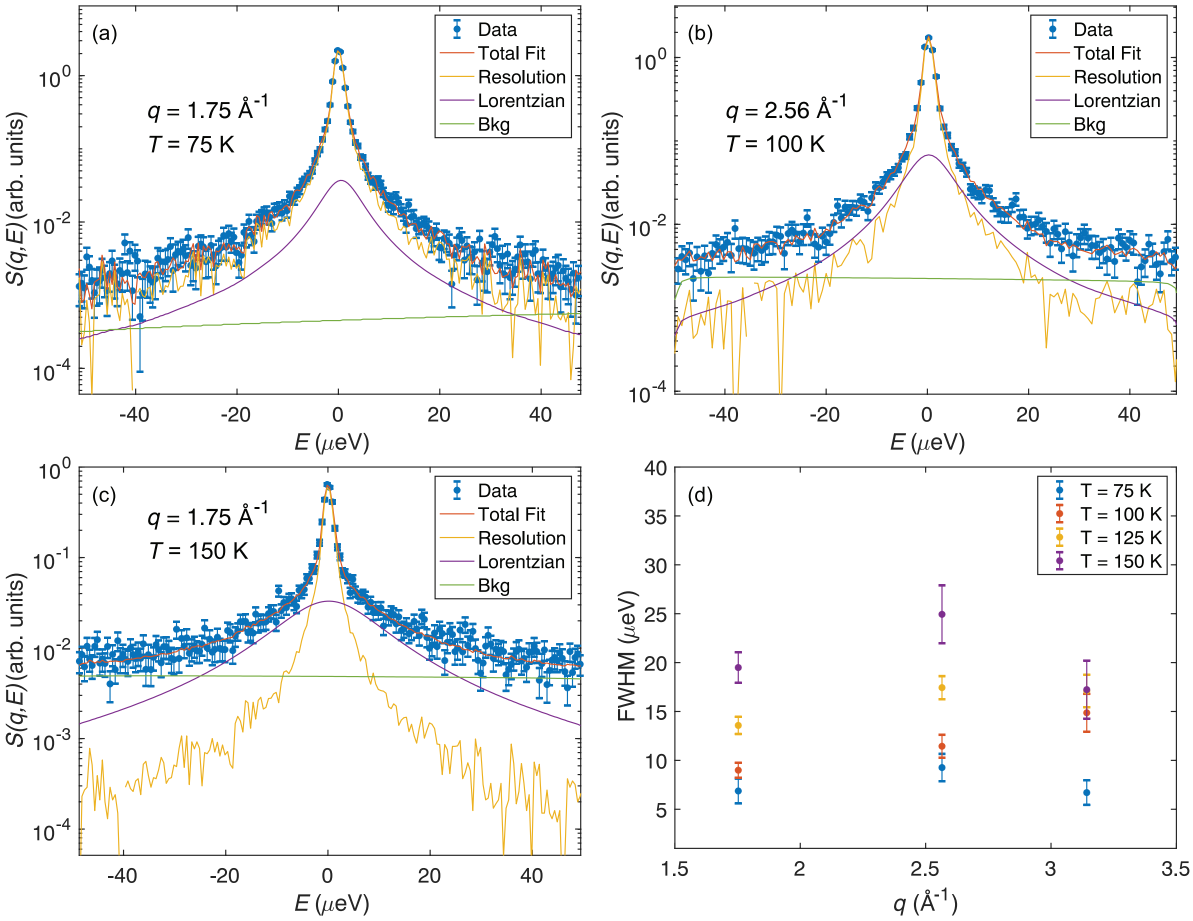

Figure S2: IN16B QENS data of $FAPbI_3$. (a-c) QENS spectra together with their fits, and (d) quasielastic linewidth.

S2. Quasielastic neutron scattering on $MAPbI_3$

We performed experiments on $MAPbI_3$ on IN16B and IN6. The results are summarized in Figure S3-S5 and are, generally, in excellent agreement with previous QENS studies of $MAPbI_3$.[S3–S7] Figure S3 shows plots of EFWS and IFWS (at 4 μeV and 10 μeV), as measured upon cooling from 300 K to 2 K on IN16B. Figure S4 shows fits of the QENS spectra at 75 K, 100 K, and 125 K, as measured on IN16B. Figure S5 shows (a) the QENS spectrum at 250 K, as measured on IN6, and (b) the result of an EISF analysis based on the IN6 data.

The EFWS reveals a marked increase at the tetragonal-to-orthorhombic phase transition around 162 K upon cooling. Upon further cooling, the elastic intensity increases more rapidly, before it levels out below about 50 K. The IFWSs show maxima at about 95 K and 110 K, respectively. In addition, the IFWS taken at 4 μeV shows a distinct feature at 170 K, which is near the tetragonal-to-orthorhombic phase transition. This latter feature is probably related to the activation of C_4 rotations of the whole MA molecule.[S4] Fits to the IFWSs yield activation energies of 48.6 meV and 51.9 meV, for the 4 μeV and 10 μeV data, respectively and the corresponding values of $2\hbar/\tau_0$ are 1.9236 meV and 2.86 meV, respectively. These values are in agreement with what is obtained from the fitting of the QENS spectra ($E_a = 48.46$ meV) (cf. Fig. S3 (b)), which suggests that the values are robust and that the dynamics of the MA cations in the orthorhombic phase are consistent with a single relaxational process. Furthermore, the activation energy of methyl/ammonia rotation of the MA cations in the orthorhombic phase is in good agreement with the values reported in previous QENS studies.[S4,S7]

The IN6 data are generally in good agreement with the results reported in an earlier study on $MAPbI_3$.[S4] Two dynamical processes of $MAPbI_3$ were observed in the tetragonal phase which correspond to 3−fold rotation of the methyl/ammonia group around the C − N axis, and 4−fold rotation of the whole MA molecule in the tetragonal crystal environment. In the orthorhombic phase the 4−fold rotation of the whole MA molecule becomes frozen, and we thus only observe a single QENS component. In the cubic phase, the 3−fold rotation of the methyl/ammonia group most likely becomes too fast to be observed on IN6, and goes out of the dynamic window of the instrument and into the background intensity.

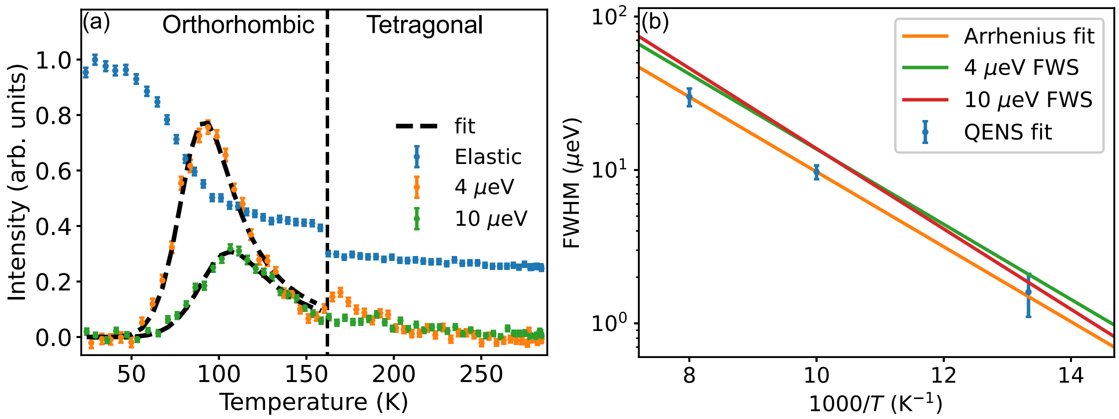

Figure S3: IN16B QENS data of $MAPbI_3$. (a) EFWS and IFWSs summed over all measured q-values. The elastic intensity is normalized to a maximum value of unity and the inelastic intensities are multiplied by a factor of 15 for increased visibility. An elastic contribution was subtracted from the inelastic intensities by determining the relative intensity at 4 μev and 10 μev in the 2 K QENS spectra. (b) Quasielastic linewidth determined from fits to the inelastic intensity and QENS spectra, respectively.

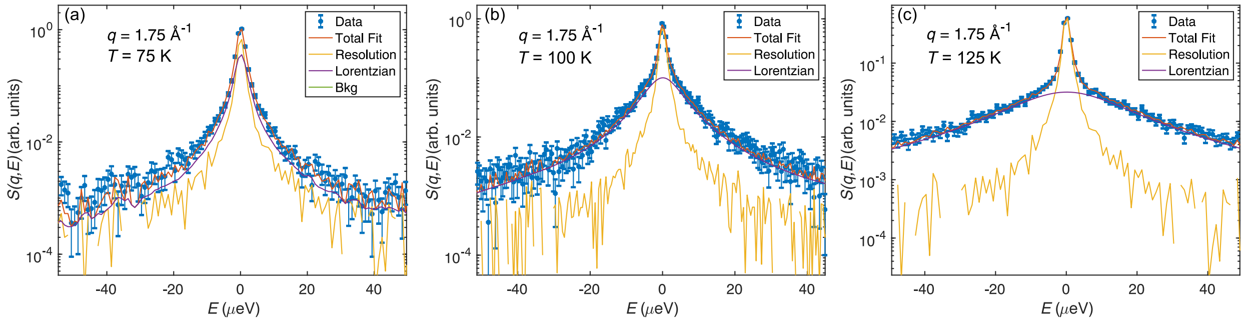

Figure S4: IN16B QENS spectra of $MAPbI_3$, together with their fits.

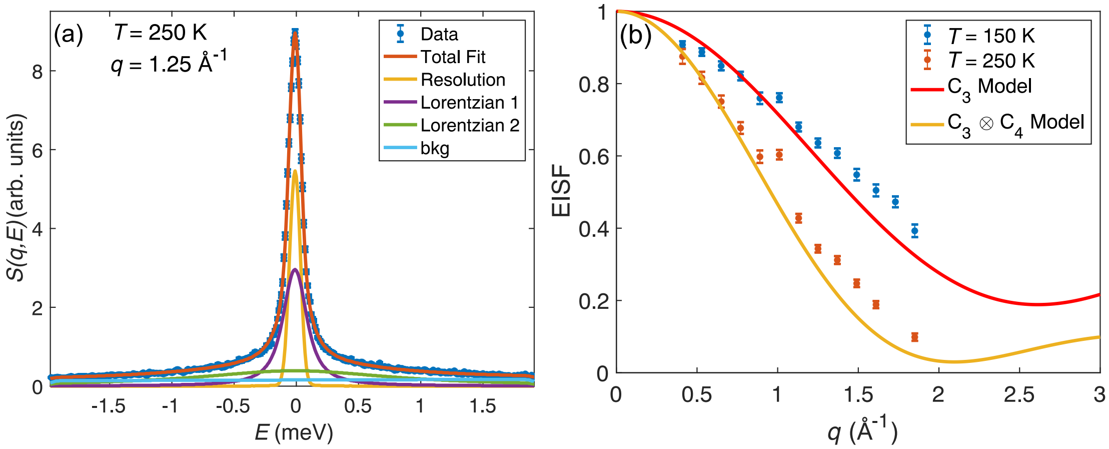

Figure S5: IN6 QENS spectrum of $MAPbI_3$, together with its fit at $T = 250$ K. (b) EISF in the orthorhombic and tetragonal phases, respectively.

S3. EISF models for $FAPbI_3$

S3.1 Cubic α-phase

The EISF model for isotropic rotational diffusion on a sphere of radius r is given by[S8]

$$\mathrm{EISF}_{\text{isotropic}} = j_0^2(q\,r), \tag{4}$$

where $j_0(x) = \sin(x)/x$ is the zeroth-order spherical Bessel function. We also considered the model where the FA cations are disordered amongst 12 different sites, where the C−H bond can point toward any of the six faces of a cube and for which each of these directions there are two directions of the N···N axis. The EISF model for this is given by

$$\mathrm{EISF}_{\text{12-site}} = \frac{1}{60}\sum_{i}^{60} j_0(q\,d_i). \tag{5}$$

Here, $d_i = |\mathbf{r}_i - \mathbf{r}_0|$ is the jump distances for the 60 different H atom sites [12 (sites) ×5 (H atoms in FA) = 60]. Note, some of these distances are zero, and some of them are the same.

S3.2 Tetragonal β-phase

Weber *et al.*[S1] showed that, in the tetragonal β-phase, the FA cations are disordered over four sites, which mainly involve rotations around the N···N axis. We considered jump diffusion between these sites, and this process will correspond to rotations around the N···N axis. Each of the three in-equivalent H atoms in FA has a different jump distance during this rotation, and the EISF is thus a weighted average of the EISF for each individual H atom.[S9] The EISF for this rotation is given by

$$\mathrm{EISF}_{\mathrm{N\cdots N}} = \frac{1}{5}\left[\mathrm{EISF}_{\mathrm{N\cdots N}}(\mathrm{H1}) + 2\times\mathrm{EISF}_{\mathrm{N\cdots N}}(\mathrm{H2}) + 2\times\mathrm{EISF}_{\mathrm{N\cdots N}}(\mathrm{H3})\right], \tag{6}$$

where

$$\mathrm{EISF}_{\mathrm{N\cdots N}}(\mathrm{H}i) = \frac{1}{4}(1 + j_0(qd_{i1}) + j_0(qd_{i2} + j_0(qd_{i3})) \tag{7}$$

is the EISF for the i:th hydrogen atom. The jump distances for each in-equivalent H atom are given by $d_{11} = 0.74$ Å, $d_{12} = 3.01$ Å, $d_{13} = 2.92$ Å, $d_{21} = 0.14$ Å, $d_{22} = 0.606$ Å, $d_{23} = 0.59$ Å, $d_{31} = 1.09$ Å, $d_{32} = 2.21$ Å, and $d_{33} = 1.92$ Å.

We also considered jump rotations around the C−H axis. In this rotation, the H1 atom is static, and the EISF is given by

$$\mathrm{EISF}_{\mathrm{C-H}} = \frac{1}{5}\left[1 + 2\times\mathrm{EISF}_{\text{C-H}}(\mathrm{H2}) + 2\times\mathrm{EISF}_{\text{C-H}}(\mathrm{H3})\right], \tag{8}$$

where

$$\mathrm{EISF}_{\text{C-H}}(\mathrm{H}i) = \frac{1}{4}(1 + j_0(qd_1) + 2j_0(qd_1/\sqrt{2})). \tag{9}$$

The jump distances are $d_1 = 3.99$ Å and $d_2 = 2.6$ Å for H1 and H2, respectively.

References

(S1) Weber, O. J.; Ghosh, D.; Gaines, S.; Henry, P. F.; Walker, A. B.; Islam, M. S.; Weller, M. T. Phase Behavior and Polymorphism of Formamidinium Lead Iodide. Chem. Mater. **2018**, 30, 3768–3778.

(S2) Fåk, B.; Dorner, B. Phonon line shapes and excitation energies. Physica B **1997**, 234, 1107–1108.

(S3) Leguy, A.; Frost, J. M.; McMahon, A. P.; Sakai, V. G.; Kockelmann, W.; Law, C.; Li, X.; Foglia, F.; Walsh, A.; O'regan, B. C., et al. The dynamics of methylammonium ions in hybrid organic–inorganic perovskite solar cells. Nat. Commun. **2015**, 6, 1–11.

(S4) Chen, T.; Foley, B. J.; Ipek, B.; Tyagi, M.; Copley, J. R. D.; Brown, C. M.; Choi, J. J.; Lee, S.-H. Rotational dynamics of organic cations in the $CH_3NH_3PbI_3$ perovskite. Phys. Chem. Chem. Phys. **2015**, 17, 31278–31286.

(S5) Li, B.; Kawakita, Y.; Liu, Y.; Wang, M.; Matsuura, M.; Shibata, K.; Ohira-Kawamura, S.; Yamada, T.; Lin, S.; Nakajima, K.; Liu, S. F. Polar rotor scattering as atomic-level origin of low mobility and thermal conductivity of perovskite $CH_3NH_3PbI_3$. Nat. Commun. **2017**, 8, 16086.

(S6) Schuck, G.; Lehmann, F.; Ollivier, J.; Mutka, H.; Schorr, S. Influence of Chloride Substitution on the Rotational Dynamics of Methylammonium in $MAPbI_{3-x}Cl_x$ Perovskites. J. Phys. Chem. C **2019**, 123, 11436–11446.

(S7) Li, J.; Bouchard, M.; Reiss, P.; Aldakov, D.; Pouget, S.; Demadrille, R.; Aumaitre, C.; Frick, B.; Djurado, D.; Rossi, M.; Rinke, P. Activation Energy of Organic Cation Rotation in $CH_3NH_3PbI_3$ and $CD_3NH_3PbI_3$: Quasi-Elastic Neutron Scattering Measurements and First-Principles Analysis Including Nuclear Quantum E. J. Phys. Chem. Lett. **2018**, 9, 3969–3977.

(S8) Bée, M. Quasielastic Neutron Scattering: Principles and Applications in Solid State Chemistry and Materials Science; Adam Hilger, Bristol, 1988.

(S9) Yildirim, T.; Gehring, P.; Neumann, D.; Eaton, P.; Emrick, T. Neutron-scattering investigation of molecular reorientations in solid cubane. Phys. Rev. B **1999**, 60, 314.
